# Rapid-onset cancer

**DOI:** 10.1016/j.tvr.2024.200312

**Published:** 2025-01-02

**Authors:** Andrea Bilger, Paul F. Lambert

**Affiliations:** McArdle Laboratory for Cancer Research, University of Wisconsin School of Medicine and Public Health, 1111 Highland Avenue, Madison, WI, 53705, USA

**Keywords:** Rapid, Slow, Onset, Cancer, Virus, Congenital, Carcinogenesis

## Abstract

Human cancers are generally thought to develop over the course of decades. Such slow progression is well documented for a variety of cancers that we designate “slow-onset” cancers. “Rapid-onset” cancers, in contrast, can develop in a matter of months in humans or in as little as 9 days in mice. These cancers often develop under conditions that might be expected to accelerate cancer development: early development, immune deficiency, or viral infection. We will discuss rapid-onset cancers in the context of the "hallmarks of cancer" – properties cells must acquire in order to become malignant – focusing on how viruses are particularly well suited to causing rapid-onset cancer.

## 1 What is “rapid-onset" cancer?

To understand what it means for a cancer to develop rapidly, we need to know what it means for cancers to develop slowly. This review will summarize some of the decades of data on cancer development in tissues where available evidence supports a multistep process that requires decades in people or several months in rodent models: “slow onset” cancers. We will then describe a variety of “rapid onset” cancers: genetically engineered, congenital, and virus-induced cancers in patients and/or animals where onset occurs in as little as months in people or weeks in animal models. Some of these rapid-onset cancers are also "early-onset" cancers – cancers that develop in young patients or animals; others – particularly virus-induced cancers – can develop rapidly even in adults.

## 2 Multistep carcinogenesis: “slow-onset cancer”

Cancer is generally considered a disease of aging, with a median age at diagnosis in the United States of 67 [1,2]. In the 1950s, analysis of cancer mortality statistics, relating the frequency of deaths from cancer to the age of those deaths, suggested that many cancers result from the accumulation of six to seven mutations [1]. A correction for mutations that confer a proliferative advantage reduced this number to 4–5 mutations per cancer cell [2]. Mutational analysis has largely validated this hypothesis for a variety of cancers, revealing an average of 4–5 cancer driver mutations – including mutations that confer a proliferative advantage – for nearly 2700 cancers of 38 types [3].

Analyses of cancer latency in cases likely initiated by exposure to chemicals such as asbestos (mesothelioma) or arsenic (bladder cancer) suggest such cancers develop over the course of decades, with a median time to onset of 40+ years [[4], [5], [6], [7]]. Analyses of lesions of increasing dysplasia, some longitudinal, have confirmed that many types of cancer evolve slowly, through the stepwise accumulation of mutations. Such multistep carcinogenesis has been described for colorectal, head-and-neck, and a variety of other cancers [[8], [9], [10], [11]].

In mice, cancers induced by chemical carcinogenesis develop months or more after carcinogen treatment. When adult mice susceptible to liver cancer (F1 hybrids of the inbred C57BL/6 and C3H/He strains) were treated with a nearly lethal dose of the carcinogen diethylnitrosamine (DEN), hepatocellular carcinomas - the most common carcinogen-induced cancer in the National Toxicology Program’s two-year bioassay - were diagnosed more than 14 months later [12]. Treating pre-weanling mice with a lower dose of DEN and assessing lesions at regular intervals reveals preneoplastic lesion development by 10 weeks and hepatocellular carcinoma by 20 weeks in susceptible males [13]. Chronic treatment with the carcinogen 4-NQO in drinking water yields oral cancers in adult mice on a similar timeline, with dysplasia developing at 8 weeks and cancer at 20 weeks or more [14].

### 2.1 The "slow-onset" colorectal cancer paradigm

Colorectal cancer (CRC) has provided a paradigm for understanding slow-onset, multistep carcinogenesis [10,11,15]. Analysis of a range of tumors from small, benign adenomas to large cancers has yielded a robust composite profile in which the first step generally consists of activation of the WNT signaling pathway (mutated in ∼93 % of CRCs), commonly via mutation of the *APC* (*Adenomatous Polyposis Coli*) tumor suppressor gene [11,15]. Later steps involve growth promotion via gain-of-function mutations in the RAS and/or PI3K signaling pathways and mutation of TP53, suppressing apoptosis and promoting invasion (Figure) [11,[15], [16], [17], [18], [19], [20]]. Mutations that disrupt DNA repair generally lead to mutations arising in the same pathways [15]. Recent DNA sequencing confirmed that the accumulation of genetic changes relevant to colon cancer correlates with time. Intestinal crypts of people from 11 to 78 years old harbor a variety of mutations, including mutations in driver genes and whole-chromosome copy number changes, that accumulate with age [21].

**Figure:**
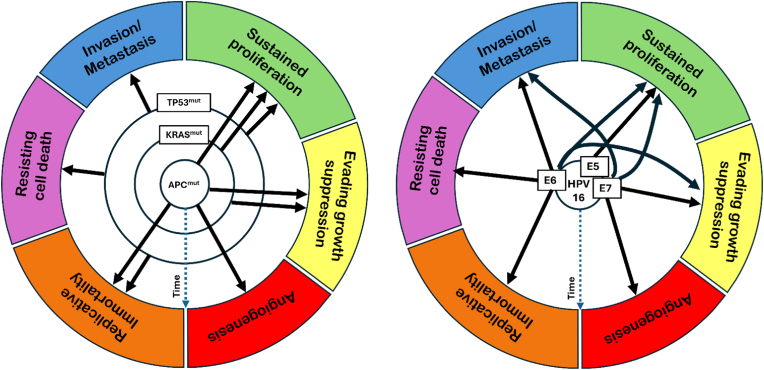
Time as a factor in acquiring the classic hallmarks of cancer in multi-step colorectal cancer relative to virally induced cancer. Left: Colorectal cancer develops over time, with mutations acquired in multiple steps [10,[16], [17], [18], [19]]. Each concentric central circle represents a critical genetic change, with larger circles representing later changes. The "Time" axis is shown as a dotted blue line. Right: The central circle represents multiple genetic changes that occur simultaneously with papillomavirus infection. High-risk human papillomaviruses such as HPV 16 express multiple oncogenic proteins – E5, E6, and E7 – soon after infection. Each of these proteins affects one or more hallmarks of cancer [22,23].

### 2.2 Germline APC mutations

The time of initial mutation for some colorectal cancers is known. Patients with Familial Adenomatous Polyposis Coli (FAP) carry a germline mutation in the *APC* gene [11], which is mutated in ∼77 % of colorectal cancers [15]. Congenital mutation of *APC* leads to the development of tens to thousands of polyps in teenagers. A subset of these tumors go on to become malignant, with an average age of colorectal cancer diagnosis of approximately 40 [24]. However, colorectal cancer has been detected in children with FAP as young as 9 [25].

Inherited mutations that cause hypermutation, such as inactivation of DNA repair genes in Lynch Syndrome patients, also accelerate colorectal carcinogenesis [11,24]. Lynch Syndrome patients develop far fewer tumors than FAP patients. This suggests that *APC* mutation more frequently initiates neoplastic growth than DNA repair mutation does, as might be expected: whereas APC directly suppresses tumor initiation via the WNT pathway, tumor initiation in response to DNA repair protein loss requires an additional step in which loss of DNA repair leads to mutation of oncogenic or tumor suppressor proteins such as APC. However, the mutator phenotype of Lynch Syndrome appears to accelerate tumor progression – resulting in a similar age of colorectal cancer diagnosis for FAP and Lynch Syndrome patients [11,24].

Mutation of *Apc* in mice causes intestinal polyposis much like that seen in FAP patients [26]. Polyps remain benign for the first 4 months after birth but can become invasive by approximately 8 months of age [27]. The difference in time to onset between humans and mice with inherited mutations in *APC/Apc* correlates with a general difference in susceptibility to oncogenic transformation between human and mouse cells, with mouse cells transforming more easily – at least in part due to a qualitative difference in telomerase expression [28].

### 2.3 “Slow onset:” ∼40 years

From these detailed studies of colorectal carcinogenesis and the longitudinal tracking of people and animals exposed to chemicals such as asbestos, arsenic, and DEN, we have a baseline for “slow-onset cancer.” Such cancers develop over approximately 40 years in people, whether initiated due to mutations present at birth in the case of FAP and Lynch Syndrome patients, or later in life due to chemical exposure. In mice, comparable mutations lead to malignancy in approximately 6 months.

## 3 Rapid-onset cancer

While many cancers evolve over decades, others develop remarkably rapidly – in as little as months in people or weeks in animals. Cancer can be induced rapidly by engineering cells to carry the same set of driver mutations identified in slow-onset cancers. But rapid-onset cancers can also arise more naturally. Some develop in the presence of factors that would be expected to accelerate carcinogenesis based on their ability to affect multiple hallmarks of cancer simultaneously, such as embryonic development or viral infection. Others develop in the event of immune deficiency, which eliminates the need to evade immune destruction – one of the emerging hallmarks of cancer [19]. Yet others involve combinations of these factors.

### 3.1 Genetically engineered cancers

Many species, from zebrafish to pigs, have been engineered to carry transgenic oncogenes and/or mutated tumor suppressors that are known to drive slow-onset cancers [[29], [30], [31], [32], [33], [34]]. Eliminating the requirement for time to accumulate these changes dramatically accelerates carcinogenesis [29,31,33,34].

A particularly rapid-onset adult mouse model combines several changes known to occur in cervical cancer: expression of the viral oncogenes HPV16 *E6* and *E7*, activating mutation of the cellular proto-oncogene *Kras*, and knockout of the tumor suppressor gene *Pten* [29,35]. These changes require excision of floxed "stop" sequences knocked into the endogenous, mutated *Kras* locus and inserted in an HPV *E6* and *E7* transgene driven by the *Keratin 14* promoter, and excision of a floxed exon of *Pten* (Table). These mutations can be induced nearly simultaneously by delivery of adeno-*Cre* to the cervicovaginal mucosa of adult mice. Mice treated with adeno-*Cre* become moribund due to invasive cancer as soon as 23 days after treatment [29] (personal communication). These results indicate that these 4 genetic changes are sufficient to cause malignancy without a time lag. Heterozygosity of *Pten*, or the absence of *E6* and *E7* or mutated *Kras*, delayed tumor development by one to several months. The longest delay in visible tumor development, 9 months, was caused by absence of E6 and E7 expression [29].

**Table:** 1Rapid-onset cancer.

Category	Cancer	Species	Age	Average or modal age at diagnosis	Earliest diagnosis found in literature	Frequent genetic changes	Frequent spontaneous regression	References
Genetically Engineered	Cervicovaginal	Mouse	Adult	53 days post induction^a^	23 days post induction^a^	Induced HPV16 *E6*, *E7*, *Kras*^*G12D*^; *PTEN*^*−/−*^	No	[29]
Genetically Engineered	Mammary	Mouse	Pup/Adult	8–12 weeks	∼21 days	MMTV-PyMT; Chr. 11 amplification; Ptprh mutation	No	[[36], [37], [38]]
Congenital/Pediatric	Retinoblastoma (bilateral)	Human	Infant	12–14 months	20 day old infant	*RB*^*−/−*^*; MYCN* amplification	No	[47,48]
Congenital/Pediatric	Neuroblastoma	Human	Infant	17 months	4 day old infant	MYCN amplification/overexpression	Yes	[50,51,102,103]
Congenital/Pediatric	Rhabdomyosarcoma	Human	Fetus/Child	<10 years	20 week fetus	Maternal chr. 11p loss	No	[62,63]
Congenital/Pediatric	Melanoma	Human	Child	12 years	6 week old infant	MAPK, PI3K pathway mutations (*N/H/KRAS*, *BRAF, NF1*)	Yes	[[65], [66], [67], [68],^70^]
Congenital/Pediatric	Melanoma	Pig (MeLiM)	Piglet	0–2 months	Birth	Candidates on Chr 2,5 7, 8, 16	Yes	[67]
Marek's Disease Virus	T-cell Lymphoma	Chicken	Chick	32–50 days. post infection	16 days post infection	ND	No	[85,104]
Murine Leukemia Virus	Myeloid Leukemia	Mouse	Adult	38 days	9–15 days post infection	ND	No	[81,83]
Mouse Papillomavirus	Squamous Cell Carcinoma	Mouse	Adult	2 weeks post infection	2 weeks post infection	ND	Yes	[89]
EBV	B-cell Lymphoma	Human	Immune-suppressed Adult	4.3 months post transplant	2 months post transplant	ND	No	[91]
EBV	B-cell Lymphoma	Human cord blood cells in Mouse	Immune-deficient Adult	28–35 days post infection	28 days post infection	ND	No	[92]

ND: Not Determined.

a Age when moribund.

Similarly, simultaneous expression of the three most frequent mutated genes found in human colorectal cancer - *Apc*, *Kras*, and *Tp53* - causes rapid-onset cancer in mice [17]. Organoids of engineered cells carrying mutant *Apc*, inducible mutant *Kras*, and inducible mutant *Tp53*, implanted orthotopically in the mouse colon, yield stage 1 cancers within 4 weeks of Cre-mediated mutant KRAS and TP53 induction [17].

A third rapid-onset model relies entirely on the expression of a single viral gene encoding the mouse polyomavirus middle T antigen (PyMT), which acts as a scaffold signal adaptor and activates SRC and SRC-like tyrosine kinases [36] (Table). Mouse PyMT expressed constitutively can induce tumors of a variety of cell types, including mammary cancers when PyMT is expressed from a mouse mammary tumor virus promoter [[36], [37], [38]]. Cancers induced in this model frequently show Chromosome 11 amplification, which is associated with PI3K pathway activation due to *ERBB2* amplification [37]. The vast majority of *MMTV-PyMT-*induced tumors also carry mutations in the *Ptprh* gene that lead to constitutive activation of EGFR [37]. PyMT-induced mammary cancers can develop as early as 3 weeks after birth [38].

### 3.2 Congenital and perinatal cancers

Approximately 130 malignant solid tumors are diagnosed in fetuses or newborns in the United States each year [39]. Some of these cancers are unique to early development, requiring transformation of a transient cell type, while others involve earlier onset of cancers also diagnosed in adults. While critical tumor suppressors have been identified for many of these cancers, including retinoblastoma and rhabdomyosarcoma, the details of their genetic/epigenetic routes to carcinogenesis are generally not well understood [40,41]. However, it is clear that the sets of mutations that drive pediatric cancers only partially overlap with those that drive adult cancers [40,42,43]. Here we review a selection of rapid-onset cancers comprised of congenital and perinatal cancers notable for their historic importance in cancer genetics (retinoblastoma), frequency (neuroblastoma), particularly early onset (rhabdomyosarcoma), spontaneous regression (neuroblastoma, melanoma), and spontaneous occurrence in both people and robust animal models (melanoma).

#### 3.2.1 Retinoblastoma (humans)

Analysis of retinoblastoma genetics gave rise to the concept of a tumor "suppressor" [44]. Retinoblastoma is a cancer of the retina, most likely derived from cone cell precursors [45,46]. The median age at diagnosis of familial, bilateral retinoblastoma is 12–14 months in the US and Canada but can be as early as 20 days [47,48] (Table). Retinoblastoma develops due to mutation of the *Retinoblastoma* (*RB1*) gene and consequent loss of RB protein, which regulates the cell cycle. Retinoma is a benign precursor of retinoblastoma that involves disruption of both alleles of the *RB1* gene [49]. Loss of RB protein is therefore not sufficient to cause malignancy. Retinoblastomas carry additional mutations, such as the amplification of Chromosome 2p carrying *MYCN*. Knockdown of *MYCN* restricts the growth of retinoblastoma cells [45,49].

Familial retinoblastoma appears analogous in some ways to colorectal cancer in FAP patients: both cancers are initiated by an inherited, heterozygous mutation in a tumor suppressor gene. Both involve the loss of the remaining allele in the development of a benign tumor. However, lesions can become malignant by 20 days of age in retinoblastoma patients, while FAP patients rarely develop colorectal cancer as early as 9 years of age. The reason for this difference in the rapidity of cancer onset has not been determined. Cone cell precursors in the retina may be particularly susceptible to carcinogenesis caused by the loss of RB because of elevated expression of MYCN and MDM2 and downregulation of p27 (CDKN1B) with cone cell maturation [46].

#### 3.2.2 Neuroblastoma (humans)

Neuroblastoma is a cancer of the sympathetic nervous system, typically occurring in the adrenal medulla and the paraspinal sympathetic ganglia [50,51]. Neuroblastoma is the most common cancer diagnosed in children under 1 year old [52]. Many neuroblastomas, including Stage IV, are diagnosed in children 0–5 months old [53] (Table). Very young patients (<1 year) are disproportionately likely to have a special form of Stage IV cancer designated Stage IVS (“S” for “Special”), in which infants have tumors in multiple tissues [51,53]. Notably, these Stage IVS cancers generally regress spontaneously. Spontaneous regression might be due to a reversal of replicative immortality caused by epigenetic reduction of telomerase activity [50,51]. High-risk neuroblastomas that do not regress frequently carry rearrangements of the *TERT* gene, with concomitant increased expression and activity of the encoded telomerase [54,55].

Neuroblastoma development is still poorly understood: no minimal set of drivers has been defined [50,51]. Most neuroblastomas amplify *MYCN* or delete miRNAs that bind to *MYCN* mRNA, suggesting that MYCN is a key driver. Expression of the pluripotent regulator LIN28B, which increases MYC expression, can initiate neuroblastoma development in transgenic mice [56]. Mutations in *ALK* (*Anaplastic Lymphoma Kinase*) are associated with familial neuroblastoma, but this gene is rarely mutated somatically [50,51]. Expression of Sonic Hedgehog (SHH) pathway mRNAs is also frequently upregulated. Chromosome 17q is amplified in the vast majority of neuroblastomas, and 11q, carrying a negative regulator of MYCN expression, is lost in about half. However, 10–30 % of neuroblastomas have no large-scale genetic aberrations, somatic mutations are infrequent, few genes are recurrently mutated, and – for high-risk neuroblastomas in older children – there is no correlation between age at diagnosis and mutation frequency [50,51,57]. These observations raise an alternative explanation in which epigenetic abnormalities during development might be involved in the initiation of neuroblastoma tumorigenesis [50,51,57]. Alternatively, cancer development might involve mutation of micro-protein-coding open reading frames not previously recognized as genes, as has recently been shown for medulloblastoma [58].

#### 3.2.3 Rhabdomyosarcoma (humans)

Rhabdomyosarcomas are cancers that appear to derive from muscle, with a median age of onset of 15 years [59]. However, some of these cancers develop within weeks or months in utero (Table). In one case, a fetus with no visible tumor at 28 weeks was found to have a 12 × 5.4 cm tumor at 33 weeks and died with diffusely spread tumor by 34 weeks of gestation [60]. In a second case, a tumor was detected in an 18-week fetus during a routine ultrasound. When the pregnancy was terminated 2 weeks later, the fetus was diagnosed with embryonal rhabdomyosarcoma [61].

Embryonal rhabdomyosarcoma, found almost exclusively in patients less than 10 years old, has a different genetic profile than the alveolar subtype found mostly in patients over 10 [62]. Over 60 % of both types of rhabdomyosarcoma have lost the maternal chromosome 11p (most with duplication of the paternal chromosome), including the imprinted region on 11p15.5 that carries the maternally expressed tumor suppressor gene *H1*9 that encodes a regulatory untranslated RNA [63]. The alveolar subtype as a rule involves a gene fusion (*PAX3-FOXO1* or *PAX7-FOXO1*) that is diagnostic and oncogenic, but not sufficient for tumorigenesis *in vivo* (shown for the more potent *PAX3-FOXO1* fusion) [64]. Small subsets of embryonal rhabdomyosarcomas have mutations such as activated *RAS* genes or other known drivers; however not all do [62,63]. This suggests that the large-scale genomic aberrations that are more prevalent in the embryonal subtype than in the alveolar subtype, such as amplification of multiple chromosomes, are likely to be critical in the development of rapid-onset rhabdomyosarcomas [62].

#### 3.2.4 Melanoma (human, pig)

Childhood melanomas have an average onset of about 12 years; however, some develop within the first year of life [65,66] (Table). These infancy-onset melanomas often arise from large congenital melanocytic lesions (nevi) that derive from neural crest cells, develop in utero, and differ histopathologically from adult melanomas [65,67,68]. Most of these congenital precancerous lesions affect the MAPK pathway, carrying activating *NRAS* (80 %) or *BRAF* (10 %) mutations. Nevi that develop postnatally carry these same mutations in inverse proportion (5 % and 80 %, respectively). Some nevi carry neither *NRAS* nor *BRAF* mutations but instead involve gene fusions that lead to overexpression of part of the *ALK* gene also associated with familial neuroblastoma [50,51,65,67,68].

One particularly aggressive case of melanoma was detected at 6 weeks of age. This cancer involved a *ZEB2:ALK* fusion gene, as well as amplifications of parts of chromosomes 6p and 11q that are frequently seen in melanoma in addition to other chromosomal aberrations [66]. *ZEB2* (*zinc finger E−box-binding homeobox 2*) is often highly expressed in melanomas and is a 5′ fusion partner in other chimeric oncogenes in a variety of cancers [66]. In the 6-week-old infant with melanoma and a second case occurring at 10 months, a *ZEB2:ALK* fusion were found in associated benign tumors, up to 10 months before malignancy was detected. This suggests that the fusion is not sufficient for malignancy. No additional somatic sequence changes were detected in these infant melanomas, suggesting that some of the other chromosomal aberrations that were found - amplifications and losses - or epigenetic factors were critical factors in progression [66].

Melanoma-bearing Lipichov Mini-pigs (MeLiM) and the related Sinclair mini-pigs also develop melanoma congenitally, as in humans, with diagnosis at birth or within the first 2 postnatal months [67] (Table). These melanomas resemble their human counterparts histopathologically and immunohistochemically, and they can metastasize to a variety of organs [67]. A number of chromosomal alterations, but no confirmed driver mutations, have been identified [67]. Notably, as is true of neural-crest-cell-derived neuroblastomas, these congenital cancers frequently regress spontaneously - even when metastatic [67,69]. Melanoma regression in pigs correlates with early macrophage infiltration and later T-cell infiltration [67,69]. Regression usually results in depigmentation of the entire animal, indicating that both normal and cancerous melanocytes are targeted. Depigmentation also correlates with spontaneous or immunotherapeutic regression in humans, where regression correlates with an increase in T cell infiltration - particularly CD8^+^ cytotoxic T cells - as in pigs [69,70]. Regression generally occurs between the second and sixth months, with temporary relapses frequently occurring before complete regression in the MeLim model [67].

### 3.3 Viral infection

The seven known oncogenic human viruses, including EBV (Epstein-Barr virus) and HPV (Human Papillomavirus), are increasingly recognized for their ability to induce multiple hallmarks of cancer [19,22,23,[71], [72], [73], [74]]. Oncogenic viruses express proteins that, among other functions, inhibit apoptosis; enable immune evasion; induce angiogenesis; promote inflammation; cause immortalization; induce chromosomal instability and epigenetic reprogramming; promote invasion and metastasis; induce metabolic reprogramming; and increase proliferation (Figure) [23,[71], [72], [73], [74], [75]].

Oncogenic viruses induce multiple hallmarks of cancer at least in part by targeting multiple tumor suppressors and proto-oncogenes [22]. Examples include: HPV16 proteins E5, E6, and E7 promoting proliferation via the EGFR signaling pathway and inactivating the tumor suppressors TP53 and RB (Figure) [22,76], and EBV proteins LMP-1 and LMP-2 suppressing apoptosis via NFKB and promoting proliferation via the PI3K and MAPK signaling pathways [22].

It seems logical that if viruses can target multiple tumor suppressors and oncogenes, inducing multiple hallmarks of cancer, they should also accelerate carcinogenesis relative to the multistep slow-onset model (Figure). Some data argue against such acceleration: while the median age of HPV infection that leads to cancer is estimated to be 21 years, the median age at which cancer is detected, depending on the tissue, is 50–68 years [77,78]. This 30–50 year lag time matches that of slow-onset cancers. However, observations in both animals and people indicate that viruses can cause cancer much more rapidly.

Below, we describe notable examples from decades of publications on rapid-onset virus-associated cancers. Much of the literature describing these cancers involves experimental animals, where the time of infection can be determined precisely and even very small lesions can be analyzed to assess malignancy. Whereas slow-onset, chemically induced malignancy in small animals requires 4 months or more (see above), virus-induced cancers can develop within little over a week. Many of these animal studies, as well as cases of human patients with rapid-onset viral cancer, have involved infection under conditions where one of the hallmarks of cancer might be achieved without mutation, with developing tumors benefitting from the rapid proliferation associated with embryonic or early postnatal tissue growth, the natural invasiveness of infected immune cells, or the susceptibility of immune-deficiency [36,[79], [80], [81]].

#### 3.3.1 Murine leukemia virus (mouse)

Murine Leukemia Viruses (MLVs) are retroviruses found as integrated proviruses in most inbred mouse strains [82]. In what might be the most rapid documented onset of cancer, leukemia filtrate from the RF/Up strain caused myeloid leukemia in adult mice as soon as 9 days post intravenous injection [81] (Table). Injection of virus derived from the RFM/Un strain yielded comparably rapid-onset leukemias (as early as 15 days post infection; Table) [83].

MLV carcinogenesis has been ascribed to viral integration near cellular proto-oncogenes, which are then expressed at higher levels, and to the MLV P50 protein that regulates transcription from the MLV promoter and cellular genes [84]. P50 can promote proliferation and growth independence while suppressing apoptosis [84]. MLV-induced leukemias might also benefit from myeloid cells’ natural ability to enter tissues, becoming malignant more rapidly because they need not acquire mutations or epigenetic changes to achieve the invasiveness that is a hallmark of cancer.

#### 3.3.2 Marek’s disease (chicken)

Another striking example of particularly rapid onset viral cancer is Marek’s disease, an avian cancer caused by a herpesvirus: Marek’s Disease Virus (MDV) [85]. Marek's disease has a historic role in cancer prevention, as vaccines first developed in the 1970s - pioneering anti-cancer vaccines - have mostly controlled transmission and cancer in chicken flocks. MDV infection is associated with permanent immune suppression in susceptible chicken strains or in infections involving highly oncogenic virus strains. Chicks infected with MDV develop gross T-cell lymphomas in as little as 16 days post infection [80] (Table). Lymphomas can also develop in adults of susceptible strains [86].

#### 3.3.3 MmuPV1 (mouse)

The study of papillomavirus-induced cancer was transformed with the discovery, reported in 2011, of papillomavirus MmuPV1 in the cutaneous warts of immune-deficient mice [87,88]. This virus can also infect immune-competent laboratory mice, inducing cancer in both cutaneous and mucosal squamous epithelial tissues, in the presence or absence of cofactors such as carcinogens or estrogen. Squamous cell carcinoma (SCC) is generally diagnosed 4–6 months post infection [88].

Recently, however, MmuPV1-induced SCC was shown to arise within 2 weeks of infection - more rapidly than any previously reported epithelial cancer [89] (preprint; Table). These rapid-onset SCCs developed following infection of adult, immune-competent FVB/N mice, in tissues prone to papillomavirus-associated SCC in human patients such as the base of the tongue and the anus. Remarkably, these invasive tongue and anal cancers regressed completely, spontaneously, within 2–6 weeks. T cells, implicated in the spontaneous regression of childhood cancers and neonatal swine melanoma described above, were required for this regression. However, tumors in the skin, where this virus was originally found, only regressed partially [89] (preprint). These results indicate that tumor cell invasion can precede the ability to evade the immune system. The factors responsible for these rapid-onset cancers and their differential susceptibility to regression have not been identified.

#### 3.3.4 EBV (human)

EBV, which is associated with 2 % of human cancers, infects and causes tumorigenesis in lymphocytes as well as epithelial cells [73,90]. In the specialized context of iatrogenic immune suppression associated with organ transplantation, cases of lymphoma have been diagnosed less than 2 months post apparent primary infection of an EBV-negative patient by tissue from an EBV-positive donor [91]. Similarly, in immune-deficient mice, EBV-infected, CD34-depleted human cord blood injected into the peritoneum yields lymphomas in as little as 4 weeks [92].

#### 3.3.5 HPV (human)

HPVs are associated with approximately 5 % of human cancers [73]. Like MmuPV1 and other papillomaviruses, HPVs infect and cause tumors of squamous epithelium [22,23,74]. The majority of HPV-associated malignancies worldwide are cancers of the cervix, the fourth most common cancer [23]. HPV is associated with over 95 % of cervical cancers, which generally develop over decades after sexually transmitted HPV infection [73,77,78]. However, some cervical cancers develop much more rapidly, with diagnosis in women in their late teens and early twenties [93]. This apparent rapid onset is corroborated by the observation that 10–25 % of invasive cervical cancers develop within 1–5 years of a normal cytological screen, particularly in younger women [93,94]. Development of rapid-onset cervical cancers is not strongly linked to HPV genotype or to other assessed risk factors such as number of pregnancies, contraceptive use, or smoking [93]. Younger women are more likely to have rapid-onset cervical cancer as assessed by pap smear [93,94]. Rapid-onset cases far more frequently involve a glandular component compared with normal-onset cases. The multipotent progenitors that can give rise to both squamous epithelium and glandular epithelium lie in the cervical transformation zone [95]. These multipotent progenitors may have more stem-like properties [95] and may therefore require fewer genetic changes to achieve the hallmarks of cancer.

Oropharyngeal squamous cell carcinoma (OPSCC) has overtaken cervical cancer in the United States as the most common HPV-associated malignancy, with 70 % of OPSCCs testing positive for HPV [96]. Oropharyngeal cancers are often detected only after they have metastasized to local lymph nodes, and the corresponding primary cancers are often small and notoriously hard to find [[97], [98], [99]]. Robotic surgery has been used to detect initially unknown primary cancers at the base of the tongue, some as small as 2 or 3 mm [97,98]. These small cancers might reflect rapid progression from initiation to metastasis. Consistent with this possibility, circulating tumor DNA (ctDNA) analysis suggests that OPSCC is often diagnosed long after onset [100] (preprint). Blood samples collected prospectively from patients up to 10 years before OPSCC diagnosis revealed circulating tumor DNA (ctDNA) sharing the cancer's mutational signature [100]. Such early cancer development could be explained by recent computer analysis of HNSCC mutations that infers the timing of progression [101]. This modeling suggested that key drivers of HPV+ OPSCC and other head-and-neck cancers, such as HPV integration and *PIK3CA* amplification, occur 20–30 years before diagnosis [101]. These results suggest that development of some HPV-associated HNSCCs might involve rapid onset.

## 4 Conclusion

While slow-onset cancers develop over decades in people and over 4 months or more in rodents, rapid-onset cancers develop one to two orders of magnitude faster. These cancers grow quickly, invade, and can metastasize. However, a subset regress spontaneously - apparently failing to acquire robust hallmarks of cancer such as replicative immortality or evasion of the immune system. While rapid-onset cancers require some of the same factors required for slow-onset cancers, critical cellular, genetic or epigenetic factors responsible for the rapidity of their onset have yet to be determined.

## CRediT authorship contribution statement

**Andrea Bilger:** Writing – review & editing, Writing – original draft. **Paul F. Lambert:** Writing – review & editing.

## Declaration of competing interest

The authors declare that they have no known competing financial interests or personal relationships that could have appeared to influence the work reported in this paper.

## Data Availability

No data was used for the research described in the article.
